# Functional correlates of Apolipoprotein E genotype in Frontotemporal Lobar Degeneration

**DOI:** 10.1186/1471-2377-6-31

**Published:** 2006-08-24

**Authors:** Barbara Borroni, Daniela Perani, Silvana Archetti, Chiara Agosti, Barbara Paghera, Giuseppe Bellelli, Monica Di Luca, Alessandro Padovani

**Affiliations:** 1 Centre for Aging Brain and Neurodegenerative Disorders, Department of Neurology, University of Brescia, Italy; 2 Vita-Salute H San Raffaele University, IRCCS H San Raffaele, Institute of Bioimaging and Molecular Physiology, Milan, Italy; 3 Department of Laboratories and Diagnostics, University of Brescia, Brescia, Italy; 4 Nuclear Medicine, Brescia Hospital, Brescia, Italy; 5 Geriatric Unit, "Ancelle della Carità" Hospital, Cremona, Italy; 6 Centre of Excellence for Neurodegenerative Disorders and Department of Pharmacology, University of Milan, Italy

## Abstract

**Background:**

It has been recently demonstrated that in Frontotemporal Lobar Degeneration (FTLD) memory deficits at presentation are commoner than previously thought. Apolipoprotein E (ApoE) genotype, the major genetic risk factor in sporadic late-onset Alzheimer Disease (AD), modulates cerebral perfusion in late middle-age cognitively normal subjects. ApoE ε4 homozygous have reduced glucose metabolism in the same regions involved in AD.

The aim of this study was to determine whether ApoE genotype might play a key-role in influencing the cerebral functional pattern as well as the degree of memory deficits in FTLD patients.

**Methods:**

Fifty-two unrelated FTLD patients entered the study and underwent a somatic and neurological evaluation, laboratory examinations, a brain structural imaging study, and a brain functional Single Photon Emission Tomography study. ApoE genotype was determined.

**Results:**

ApoE genotype influenced both clinical and functional features in FTLD. ApoE ε4-carriers were more impaired in long-term memory function (ApoE ε4 vs. ApoE non ε4, 6.3 ± 3.9 vs. 10.1 ± 4.2, p = 0.004) and more hypoperfused in uncus and parahippocampal regions (x,y,z = 38,-6,-20, T = 2.82, cluster size = 100 voxels; -32,-12,-28, T= 2.77, cluster size = 40 voxels).

**Conclusion:**

The present findings support the view that ApoE genotype might be considered a disease-modifying factor in FTLD, thus contributing to define a specific clinical presentation, and might be of relevance for pharmacological approaches.

## Background

Frontotemporal Lobar Degeneration (FTLD) is a complex neurodegenerative syndrome whose broad phenotype is characterised by personality changes, behavioural disturbances, impairment in language, and in executive functions [1].

Memory deficits are not considered prominent characteristics at disease onset, but more recent studies demonstrated that amnesia at presentation in pathologically proved FTLD cases is commoner than previously thought [2]. Notwithstanding, the underlying basis of such impairment is still unexplained.

Apolipoprotein E (ApoE) is established as the major genetic risk factor in sporadic late-onset Alzheimer Disease (AD), which is mainly characterised by memory impairment [3]. Moreover, it has been demonstrated that ApoE genotype modulates cerebral perfusion in late middle-age cognitively normal subjects, ApoE ε4 homozygous having reduced glucose metabolism in the same regions involved in AD [4].

Based on these observations, we hypothesised that ApoE genotype might play a key-role in influencing the cerebral functional pattern as well as the degree of memory deficits in FTLD patients. To this aim, FTLD patients were enrolled, ApoE genotype defined, and neuropsychological standardized assessment as well as Single Photon Emission Tomography (SPECT) cerebral perfusion patterns assessed.

## Methods

### Subjects

This is a cross-sectional study of patients consecutively recruited from a large sample assessed at the Centre for Aging Brain and Neurodegenerative Diseases, University of Brescia, Italy.

All patients fulfilled international consensus criteria for FTLD [5-7]. In keeping with other studies, we retain the term FTLD as a general super-ordinate label with a subsequent subdivision into five major clinical subtypes : 1. Behavioural or frontal variant FTD (fvFTD) [7], 2. Semantic Dementia (SD) [7], 3. Progressive Non-fluent Aphasia (PNFA) [7], 4. Progressive Supranuclear Palsy (PSP) [6], and 5. Corticobasal Degeneration (CBD) [5].

All subjects performed a somatic and neurological evaluation, laboratory examinations, a brain structural Magnetic Resonance Imaging study, and a brain functional SPECT study. ApoE genotype was determined. Thirty-two out of 52 patients (60%) performed cerebrospinal fluid (CSF) Tau, Phospho-Tau and Abeta dosage to further confirm FTLD diagnosis, being Abeta dosage within the normal range and thus further excluding other kind of dementia, i.e. AD.

The diagnostic assessment included a review of full medical history, a semistructured neurological examination, including motor subscale of Unified Parkinson Disease Rating Scale, and a complete mental status evaluation by two independent and experienced neurologists; only patients with full consensus agreement by both neurologists were enrolled.

Global cognitive function assessment was made according to a standardized battery, including the Mini-Mental State Examination (MMSE) [8]. The neuropsychological assessment was made with the following tests: Raven Colored Progressive Matrices [9], Controlled Oral Word Association Test and Category Fluency [10], Clock Drawing Test [11], Rey Complex Figure Copy and Recall [12], Story Recall Test [13], Digit Span [14], Trail Making Test A and B [15], Token Test [16], and De Renzi Imitation Test [17]. Instrumental Activities of Daily Living (IADL) [18], and Basic Activities of Daily Living (BADL) [19] were assessed as well. Behavioural and psychiatric disturbances were evaluated by Neuropsychiatry Inventory (NPI) [20], and Frontal Behavioral Inventory (FBI) [21].

Stringent exclusion/inclusion criteria were applied, as follows: a) cerebrovascular disorders, previous stroke, hydrocephalus, and intra-cranial mass, documented by MRI; b) a history of traumatic brain injury or another neurologic disease; c) another extrapyramidal syndrome (e.g. Parkinson disease, Lewy Body Disease, Vascular Parkinsonism, Multiple System Atrophy), according to current clinical criteria; d) significant medical problems; e) major depressive disorder, bipolar disorder, schizophrenia, substance use disorder, or mental retardation.

Inclusion criteria: a) mild cognitive decline (Mini-Mental State Examination, MMSE ≥18), to avoid confounds in functional data analysis; b) evidence of frontotemporal hypoperfusion in single subject analysis, excluding subjects with temporo-parietal involvement mimicking AD pattern; c) CSF Abeta levels>800 pg/ml, when available.

For brain functional data comparisons, a group of healthy subjects (n = 15, mean age ± SD = 56.3 ± 15.4) were recruited, and underwent a brain SPECT study.

All participants were made fully aware about the aims of the research and the signature of an informed consent was sought from all subjects. The work was conducted in accordance with local clinical research regulations and conformed to the Helsinki Declaration.

### ApoE genotype analysis

Genomic DNA was extracted from blood samples according to standard procedures. Genetic variation at the ApoE locus was determined by restriction isotyping using FTLDR amplification and subsequent digestion with *Hha I *(*New England Biolabs*). Clinical diagnosis and SPECT analysis were performed in blind to ApoE genotype.

### ^99m^Tc-ECD SPECT acquisition and processing

Subjects were administered an intravenous injection of 1110 MBq ^99m^Tc-ECD (ethylcysteinate dimer, Neurolite, Bristol-Myers Squibb Pharma), and were imaged a dual-head rotating gamma camera (VG MILLENIUM GE), according with European procedure guidelines [22].

Basic image processing and voxel-based data analyses were performed by SPM02 routines (Wellcome Department of Cognitive Neurology, London, UK) implemented in MATLAB 6.1 (Mathworks, Sherborn, MA) [23]. SPECT images were spatially normalized by affine 12-parameter transformation onto a SPECT template conforms to the Talairach and Tournoux space. Normalized images were represented on a 79 × 95 × 68 matrix with 2 × 2 × 2 mm voxel size. To account for individual variability in structure-function relationships, an isotropic Gaussian filter was used to smooth the spatially normalized SPECT images with a FWHM of 8 mm.

### Statistical analysis

Differences between demographic and clinical characteristics according to ApoE genotype were evaluated by Mann-Whitney test and Chi-Square test, as indicated. Results are expressed as percentage or mean ± Standard Deviation. The significant level was set at *p *< 0.05.

In regard to SPECT data, global differences in the distribution of the tracer's uptake and age effect on it were covariated out for all voxels [9]. Comparisons across the different groups were made using *t*-statistics with appropriate linear contrasts. Threshold was set at *p *< 0.01 (minimum cluster size = 40 voxels).

## Results

### Subject characteristics

Fifty-five patients with the clinical diagnosis of FTLD entered the study from March 2003 through April 2005. Among them, three were subsequently excluded to avoid confounds: one because of SPECT perfusion pattern involving temporo-parietal region and cingulate areas, and two because of low CSF Abeta and high CSF Tau levels, thus resembling AD pattern.

Fifty-two unrelated FTLD patients were finally included (23 fvFTD, 3 SD, 2 PNFA, 14 PSP, 10 CBD). Overall, FTLD patients showed mild impairment (MMSE = 25.0 ± 4.1). fvFTD showed behavioural disturbances, SD and PNFA were characterized by language deficits, whilst PSP and CBD had visuo-spatial impairment.

### ApoE genotype and patients' characteristics

Seventeen FTLD patients (32.7%) carried at least one ApoE ε4 allele (9/23 fvFTD, 2/3 SD, 1/2 PNFA, 3/14 PSP, and 2/10 CBD).

The whole FTLD patient group was divided into two subgroups, according to the presence (ApoE ε4+) or the absence (ApoE ε4-) of ApoE ε4 allele. Demographic and clinical characteristics of ApoE ε4- and ApoE ε4+ patients are reported in Table 1. The two subgroups neither differed for global cognitive impairment or for IADL or BADL scores. ApoE ε4+ carriers showed worse performances in Short Story recall compared to ApoE ε4- (6.3 ± 3.9 vs. 10.1 ± 4.2, p = 0.004). Raven Colored Progressive Matrice scores were worse in ApoE ε4+ carriers, but the performance was within the normal range in both subgroups (ApoE ε4+ vs. ApoE ε4-, 19.0 ± 7.0 vs. 24.6 ± 5.5, p = 0.003).

### Perfusion pattern according to ApoE genotype

The cerebral perfusion assessment in the whole FTLD group as well as in ApoE ε4- and in ApoE ε4+ FTLD subgroups revealed a significant frontotemporal involvement compared to controls (data not shown).

**Figure 1:**
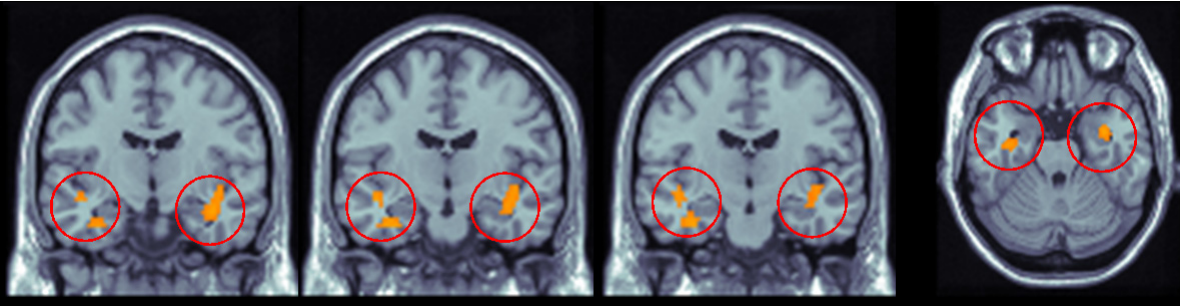
**Hypoperfusion pattern in Frontotemporal Lobar Degeneration ApoE ε4+ compared to ApoE ε4- carriers: bilateral hippocampal structure involvement**. The direct comparison of the perfusion pattern between Apolipoprotein (ApoE) ε4+ *vs*. ApoE ε4- demonstrated a significant bilateral hypoperfusion in uncus and in parahippocampal gyrus. Talairach and Tournoux coordinates. Coronal slices y = -8 to -13, axial slice z = -24,*p *< 0.01, T > 2.41, minimum cluster size = 40 voxels. Functional patterns superimposed to standard T1 weighted MRI.

The direct comparison of the perfusion pattern between ApoE ε4+ *vs*. ApoE ε4- demonstrated a significant bilateral hypoperfusion in uncus and in parahippocampal gyrus (x,y,z = 38,-6,-20, T = 2.82; -32,-12,-28, T = 2.77, see Fig. 1 and Table 2) and in medial frontal cortex (-12,12,-12, T = 3.48) in the former group. Notably, in ApoE ε4+ carriers the typical AD pattern, involving posterior parietal regions, was not found.

When the opposite comparison was considered, and the greater hypoperfusion in ApoE ε4- *vs*. ApoE ε4+ was evaluated, no voxels above the threshold were found.

In order to exclude the effect of clinical endophenotypes in determining the hypoperfusion pattern, the clinical diagnoses (fvFTD, SD, PNFA, PSP and CBD) were introduced as nuisance variable in the analysis, and the results were confirmed. Thus, we concluded that the SPECT hypoperfusion pattern was not due to a specific clinical endophenotype, but only to ApoE genotype.

## Discussion

The present study demonstrates that in a large series of mildly affected FTLD patients, ApoE ε4+ allele can influence the phenotypic expression, accounting for a greater susceptibility to uncus and parahippocampal functional impairment. Indeed, this functional pattern is associated with worse performances in test tapping long term memory functions.

It has been recently highlighted that FTLD may be characterised by memory impairment, but the possible genetic determinants of the so called amnestic FTLD have not been identified yet [2]. The autopsy-proven evidence of amnesic deficits in FTLD has suggested that pathologies mainly characterised by memory impairment, such as AD, could share the same genetic risk factors.

In AD, the role of ApoE genotype is well established [24], and it has been recently demonstrated that ApoE genotype modifies the phenotype in AD patients, the lack of ApoE ε4 directing away the pathological process from medial temporal structures [4].

ApoE genotype affects not only AD, but it may also cognitive performances in normal aging, ε4 allele-carriers showing altered memory-related cognitive processing [25]. Furthermore, cross-sectional positron emission tomography studies found that cognitively normal carriers of the ApoE ε4 allele have abnormally low measurements of the cerebral metabolic rate for glucose in the same regions as patients with AD.

No definite genetic risk factor of non-monogenic FTLD has been identified yet. Although the role of the ε4 allele of ApoE has been well established in AD, studies of ApoE allele distribution in patients with FTLD have produced variable results. Literature data on the role of ApoE genotype as a risk factor for FTLD development are controversial. Some studies have reported an increased frequency of the ApoE ε4 allele in FTLD; others have found no relationship between ApoE genotype and the risk of FTLD development [26-31]. Our results suggest higher ApoE ε4 allele frequency in FTLD (39.1%) compared to the reported incidence in healthy control subjects.

Notwithstanding, data on a possible modulating effect of ApoE genotype on functional and neuropsychological features have not been available yet. The data of the present work, if replicated in larger studies with neuropathological confirmation, may have implications for our understanding of the pathogenesis of FTLD and factors influencing the regional predilection. The fact that the ApoE ε4 genotype in our patients was associated with greater hippocampus hypoperfusion suggests the existence of common ApoE-related pathways involved in the development of different clinical FTLD subtypes.

## Conclusion

Our study carried out in a group of patients from the same geographical area and well characterized from the neuropsychological, neurological and imaging point of view suggests that ApoE genotype might be considered a disease-modifying factor in FTLD, thus contributing to define a specific clinical presentation.

## Abbreviations

FTLD: Frontotemporal Lobar Degeneration; ApoE: Apolipoprotein E; SPECT: Single Photon Emission Tomography; fvFTD: frontal variant of frontotemporal dementia; SD: Semantic Dementia; PNFA: Progressive Non-fluent Aphasia; PSP: Progressive Supranuclear Palsy; CBD: Corticobasal Degeneration; CSF: cerebrospinal fluid.

## Competing interests

The author(s) declare that they have no competing interests.

## Authors' contributions

BB conceived of the study, and participated in its design and coordination and in the draft of the manuscript; DP participated in the design of the study, in neuroimaging analyses and revised the manuscript critically for intellectual content; BP has made substantial contributions in the acquisition of the data and performed the statistical analysis; CA and GB performed the clinical evaluation of the patients and have made substantial contribution to interpretation of data; SA and MDL performed the genetic analysis and have been involved in revising it critically for important intellectual content; AP conceived of the study, and participated in its design and in coordination and drafted the manuscript. All authors read and approved the final manuscript.

## Pre-publication history

The pre-publication history for this paper can be accessed here:



## Figures and Tables

**Table 1:** Demographic and clinical characteristics according to Apolipoprotein E genotype.

***Variable***	**ApoE ε4- **(n = 35)	**ApoE ε4+ **(n = 17)	***Normal data***^	***p***^‡^
Age, years	67.1 ± 9.3	68.0 ± 6.9	-	.73
Gender, female%	51.4%	64.7%	-	.37^†^
Education, years	7.7 ± 4.2	6.9 ± 2.7	-	.99
Family History, %	17.1	17.6	-	.88
Age at onset, years	64.6 ± 8.8	65.0 ± 6.9	-	.96
Estimated disease duration, years	2.6 ± 2.2	2.3 ± 1.2	-	.94
MMSE	25.3 ± 3.9	24.4 ± 4.5	24	.54
UPDRS-III	14.7 ± 11.4	10.6 ± 10.9	0	.15
IADL, lost functions	1.2 ± 1.9	2.2 ± 2.2	0	.16
BADL, lost functions	0.6 ± 1.1	0.7 ± 1.1	0	.33
Short Story	10.1 ± 4.2	6.3 ± 3.9	7.5	.004
Rey Figure, copy	27.3 ± 6.7	23.7 ± 9.2	29	.15
Rey Figure, recall	12.5 ± 5.5	10.9 ± 7.5	15	.21
Raven Coloured Matrices	24.6 ± 5.5	19.0 ± 7.0	17.5	.003
Verbal Fluency	25.1 ± 10.7	20.6 ± 10.4	16	.13
Set Test	31.0 ± 9.3	26.6 ± 9.6	24	.13
Digit Span	5.6 ± 1.4	5.4 ± 1.7	3.75	.70
Token Test	30.2 ± 4.3	27.1 ± 5.4	29	.06
Trail Making A, ES = 0 or 1	41.0%	66.0%	>1	.48^†^
Trail Making B, ES = 0 or 1	50.0%	73.0%	>1	.44^†^
De Renzi Imitation Test	64.3 ± 12.2	66.1 ± 9.0	62	.94

ApoE: Apolipoprotein E; MMSE: Mini-Mental State Examination; UPDRS: Unified Parkinson Disease Rating Scale; IADL: Instrumental Activities of Daily Living; BADL: Basic Activities of Daily Living; ES: Equivalent Score. Neuropsychological test scores were corrected for age and education; ^ defined according to available Italian normative data. ‡ Mann-Whitney test; † Chi-Sqare test. Results are expressed as percentage or mean ± Standard Deviation. The significant level was set at *p *< 0.05.

**Table 2:** Location of the peaks of more hypoperfused areas in patients with Frontotemporal Lobar Degeneration carrying Apolipoprotein E ε4 allele.

**Area**	**Peak coordinates (mm)**			**T**	**Cluster size**
	**x**	**y**	**z**		
ApoE ε4+ vs. ApoE ε4-*					
Uncus and parahippocampal gyrus (R, L)	38	-6	-20	2.82	100
	-32	-12	-28	2.77	40
Medial frontal cortex	-12	12	-12	3.48	203

ApoE: Apolipoprotein E;

x,y, and z values localise the areas of hypoperfusion according to the MNI stereotactic coordinates. R: right, L: left
